# Challenges in Quantifying Cytosine Methylation in the HIV Provirus

**DOI:** 10.1128/mBio.02268-18

**Published:** 2019-01-22

**Authors:** Sarah A. LaMere, Antoine Chaillon, Christina Huynh, Davey M. Smith, Sara Gianella

**Affiliations:** a Department of Medicine, University of California San Diego, La Jolla, California, USA; b Veterans Affairs San Diego Healthcare System, San Diego, California, USA; Columbia University Medical College

**Keywords:** HIV latency, cytosine methylation, epigenetic silencing, non-CpG methylation

## Abstract

DNA methylation is an epigenetic mechanism most commonly associated with transcriptional repression. While it is clear that DNA methylation can silence HIV proviral expression in *in vitro* latency models, its correlation with HIV persistence and expression *in vivo* is ambiguous, particularly in persons living with HIV (PLWH) receiving antiretroviral therapy (ART). Several factors potentially contribute to discrepancies between results in the literature, including differences in integration sites, functional proviral load, sampling bias, and stochastic PCR amplification. Recent studies into genomic features of cytosine methylation sites in mammalian genes offer potentially significant insights into this mechanism. Here, we discuss the importance of these factors in the context of the HIV.

## Abstract (precis)

DNA methylation is an epigenetic mechanism most commonly associated with transcriptional repression. While it is clear that DNA methylation can silence HIV proviral expression in *in vitro* latency models, its correlation with HIV persistence and expression *in vivo* is ambiguous, particularly in persons living with HIV (PLWH) receiving antiretroviral therapy (ART).

## PERSPECTIVE

HIV latency is likely regulated through epigenetic mechanisms (1), yet few studies have evaluated epigenetic marks within and surrounding the HIV provirus (1–8). DNA methylation is one epigenetic mark that silences genes when located in promoters (9). While it is one of the best-understood epigenetic modifications (9), its role in HIV latency remains elusive. Several studies have attempted to characterize the role of DNA methylation in HIV latency (2–6), but their conclusions are inconsistent, and attempts to expound upon the findings in clinical samples from people living with HIV (PLWH) have been low yield.

Consequently, some researchers might be tempted to move away from evaluation of HIV DNA methylation, believing it plays no role in transcriptional control of HIV. While this conclusion is potentially correct, we argue that existing studies are incomplete and have failed to examine HIV proviral DNA methylation in the appropriate context, and with the appropriate tools. Many of these limitations result from a lack of available methods for specific assessment of the replication-competent HIV reservoir in clinical samples, as well as technical challenges of studying DNA methylation in the context of HIV. Here, we review and discuss the work that has been done on HIV DNA methylation and the challenges that should be overcome.

### HIV studies of DNA methylation in the literature.

Early reports suggest that DNA CpG methylation silences HIV proviral expression (8). More recent studies of CpG methylation were performed using *in vitro* HIV latency models in both cell lines and primary CD4^+^ T cells (2). While CpG methylation can silence proviral expression in latency models (2, 3), its role *in vivo* remains elusive. Studies of CpG methylation in the HIV promoter from clinical samples demonstrated that this modification is inversely correlated with viremia and proviral reactivation, suggesting that CpG methylation of the HIV promoter is a regulator of latency (3). Further evaluation has resulted in discrepancies, where long-term antiretroviral therapy (ART) is correlated with low cumulative CpG methylation in some studies (4, 6), but not in others (5). Interestingly, long-term nonprogressors and elite controllers exhibit large amounts of methylation in both the long terminal repeat (LTR) and the *env/tat/rev* CpG island (7). Together these data suggest that latency and ART-induced suppression might have different epigenetic signatures.

While there is enough evidence to suggest that CpG methylation can affect proviral expression and latency, it is difficult to correlate its presence in clinical samples with specific outcomes, such as latency. One major stumbling block is the inability to identify latently infected cells in clinical samples collected during ART, since suppression of viral replication with ART is distinct from latency.

### Evaluation of CpG islands.

Conventionally, cytosine methylation in mammals occurs in palindromic CpG dinucleotides. Two-thirds of promoters in the human genome contain clusters of CpG residues, termed CpG islands (CGIs) (9). HIV contains two highly conserved CGIs in the proximal provirus: one within the LTR, and one immediately distal to the LTR (10). Most HIV DNA methylation studies have focused upon these two CGIs, with little regard for the rest of the provirus. There is also a highly conserved CGI in the *env/tat/rev* region (ETR), though the methylation status of this region has been reported in only one study from clinical samples (6). The remainder of the HIV genome is CpG depleted (11), mirroring the human genome. This feature has been proposed to result from deamination of cytosines over time following proviral integration (12).

Early research on DNA methylation of eukaryotic genes focused on CGIs, based on methylation data for these regions in cancer cells (9). While methylation of CGIs results in transcriptional repression, the majority of promoter CGIs in somatic cells are unmethylated and regulated by other mechanisms, such as histone modifications (9). In fact, Ten-eleven Translocation (TET) enzymes, which are responsible for initiating active demethylation, are congregated around CGIs, suggesting their purpose is to keep these regions resistant to methylation. Further, many histone-modifying enzymes and transcription factors bind specifically to unmethylated CpG clusters (9).

Recent data suggested that CpG methylation plays a more prominent role in promoters without CGIs (9). For CGI-containing promoters, differential cytosine methylation exists more commonly in the regions flanking CGIs, termed CpG shores and CpG shelves (13). In the context of HIV, these regions would encompass *gag* and part of *pol*, as well as 5′ integration sites.

Only one study has examined areas outside the LTR in clinical samples using PCR-based assays (6). The areas examined in blood from 23 persons with HIV encompassed the most CpG-dense regions of the provirus, including an 898-bp region containing the LTR and a 5′ portion of *gag*, and an 1,124-bp region in the 3′ part of the virus containing parts of *nef, tat, rev,* and *env*. These regions account for only approximately 21% of the HIV genome, but contain over half (55 of 94, 58.5%) of the CpG dinucleotides, based on the HXB2 reference genome.

Underscoring the difficulty of using PCR-based methods for bisulfite sequencing of HIV in clinical samples, only 33 out of 88 DNA samples (37.5%) were successfully amplified in this study. Additionally, the authors found extremely few methylation events in the evaluated CpGs. Further, they examined the samples for unincorporated DNA and concluded that the vast majority of proviruses from their samples were from integrated DNA, suggesting that DNA methylation is unlikely to be important for HIV regulation following integration.

Unfortunately, even though this study provided the most comprehensive assessment of HIV methylation to date, the focus was limited to the areas immediately surrounding the CGI. Therefore, the most commonly differentially methylated regions typically present in CGI-containing genes (i.e., shores and shelves) still have not been assessed, either from clinical samples or from *in vitro* models.

### Non-CpG methylation.

New data recently demonstrated the presence of cytosine methylation outside CpG residues in mammalian DNA (14, 15). While these non-CpG methylation events primarily exist in embryonic stem cells, they were also described in somatic tissues, particularly in brain (15). Non-CpG methylation is mediated by the *de novo* DNA methyltransferases, i.e., DNA methyltransferase 3a (DNMT3a) and DNMT3b, which are less specific for targeting CpG dinucleotides (16). Most somatic tissues express abundant DNA methyltransferase 1 (DNMT1), which targets only hemimethylated DNA. As a result, dividing cells exhibit either little or no non-CpG methylation, as DNMT1 is more CpG specific and is the predominant DNMT in dividing cells. This is likely the reason why non-CpG methylation is localized in nondividing cells, such as neurons (16). Additionally, embryonic stem cells exhibit substantially higher expression of DNMT3a and DNMT3b, making them more prone to non-CpG methylation (16).

**FIG 1:**
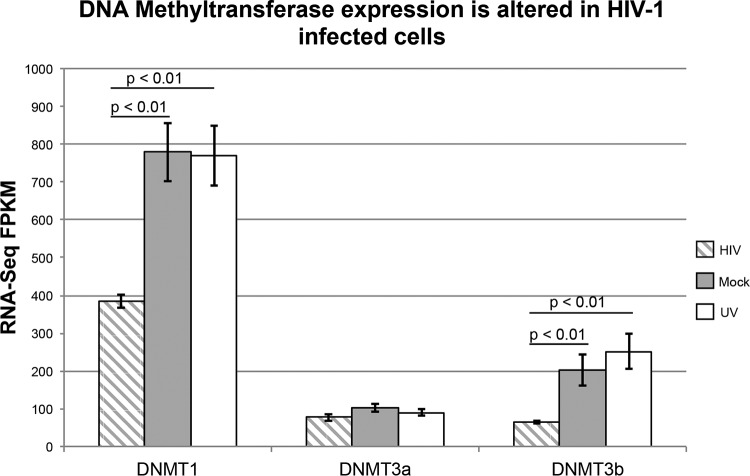
DNA methyltransferase expression is altered in HIV-1-infected cells. Data mining of RNA-Seq data from the work of Peng et al. (17) (GEO accession no. GSE53993) demonstrated that DNMT1 and DNMT3b both exhibit decreased expression in HIV-1-infected SUP-T1 cells 24 h after infection compared to mock-infected controls and cells incubated with UV-irradiated HIV-1. Error bars represent standard deviation between technical replicates. *P* values are from Mann-Whitney calculations.

Mined data from RNA-Seq expression studies (17) of HIV-infected SUP-T1 cells demonstrated that DNMT expression was dramatically altered in HIV infection (Fig. 1), and early studies reported increased *de novo* methylation in HIV-infected cells (18, 19). This could contribute to methylation outside CpG residues in HIV-infected cells experiencing *de novo* methylation of the newly integrated provirus. Additionally, non-CpG methylation was reported in the context of other exogenous retrovirus infections, such as murine leukemia virus (20, 21).

**FIG 2:**
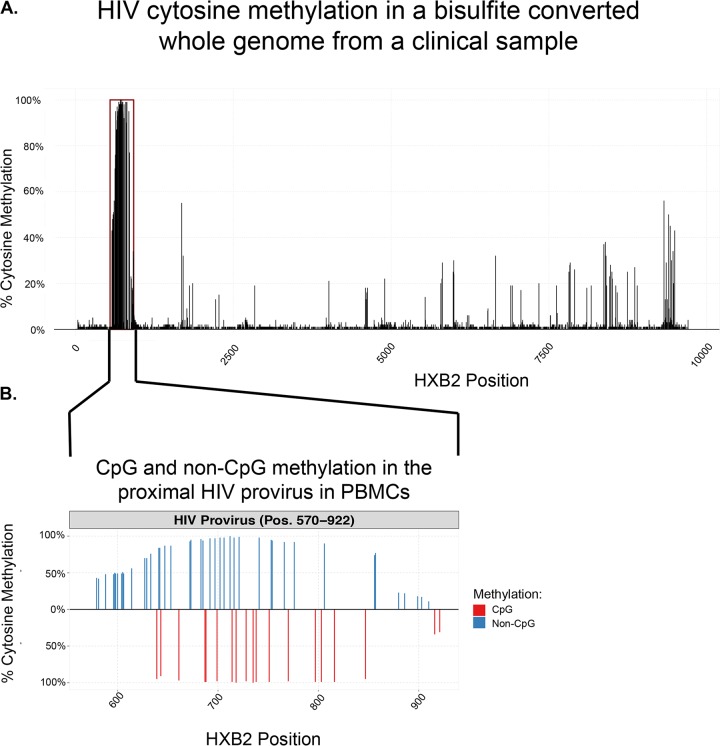
Whole-genome bisulfite sequencing of a peripheral blood mononuclear cell (PBMC) sample reveals proviral cytosine methylation. (A) Percent cytosine methylation across the entire HIV provirus. (B) A densely methylated region in the proximal provirus contains both CpG and non-CpG methylation, with the majority (68%) of methylated cytosines in non-CpG residues. As a control, lambda DNA was spiked into the sample and demonstrated a >98.8% conversion rate (see Fig. S1A and B).

So far, all studies of HIV DNA methylation have focused only on CpG methylation. In fact, some report dismissing clones where unconverted non-CpG cytosines are present (7). Part of the challenge of examining non-CpG methylation in clinical samples is that nested PCR-based methods will exclude most non-CpG methylation (16). However, whole-genome bisulfite sequencing of DNA from a clinical peripheral blood sample demonstrates that not only are there dense regions of cytosine methylation in the HIV provirus (Fig. 2A), but the majority of methylated cytosines in a densely methylated region of the proximal provirus (42 out of 61, or 68%) were methylated at non-CpG residues (Fig. 2B).

10.1128/mBio.02268-18.2 FIG S1 Quality control for whole-genome bisulfite sequencing. (A) Box plot of all unconverted cytosines across the lambda DNA control spiked into the PBMC DNA sample. (B) Distribution of unconverted cytosines across the lambda control. (C) Coverage of the lambda control. (D) Coverage of the HIV provirus. Download FIG S1, PDF file, 0.6 MB. Copyright © 2019 LaMere et al. 2019 LaMere et al. This content is distributed under the terms of the Creative Commons Attribution 4.0 International license.

### Pitfalls of PCR amplification of HIV from bisulfite-converted DNA.

**FIG 3:**
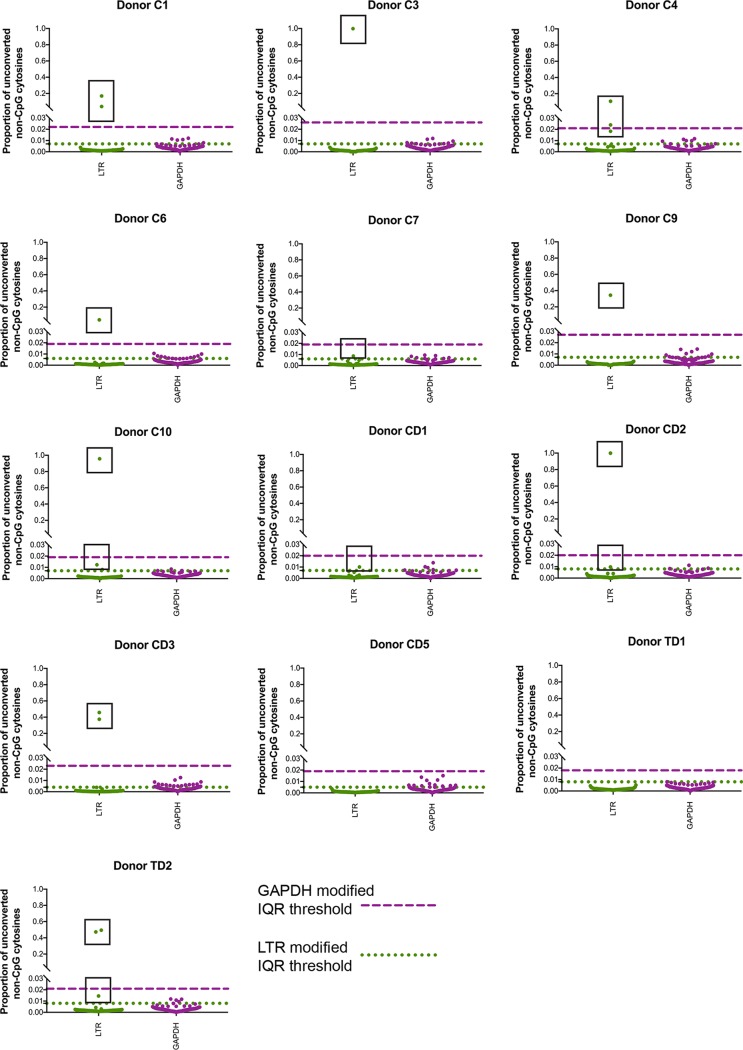
Non-CpG cytosines appear sparse when amplified with HIV-specific primers. The HIV LTR was amplified from bisulfite-converted PBMC DNA from 13 donors. Proportions of cytosine nonconversion in bisulfite-converted DNA were plotted for each cytosine across the HIV LTR amplicon (green), as well as the GAPDH intron control (purple). Each point represents a single non-CpG cytosine. A modified IQR threshold was calculated from each amplicon using the equation 7 × IQR[1 + 0.1 × log(*n/*10)] (27). The green dotted line represents a modified IQR threshold of outliers for non-CpG cytosine conversion in the HIV LTR, while the purple dashed line represents a modified IQR threshold for GAPDH. Boxes have been placed around points that exceed the threshold in the HIV LTR, representing identified non-CpG cytosine methylation.

During active replication, HIV mutates rapidly within a person (22). Therefore, designing specific PCR primers that amplify all HIV targets from clinical samples is challenging. Additionally, designing primers for bisulfite-converted DNA requires longer oligonucleotides to achieve appropriate annealing temperature and specificity, exacerbating the issue of biased amplification from highly variable sequences (23). Some variants will likely be missed, reducing the chance that all methylation events are recorded. This becomes particularly difficult in the context of highly methylated non-CpG cytosines (Fig. 2), since all cytosines in the primers need to be degenerate to avoid bias against methylation. Therefore, primers designed without degenerate bases at all cytosine positions will likely miss the majority of the highly methylated regions, resulting in artifactually sparse methylation (Fig. 3).

**FIG 4:**
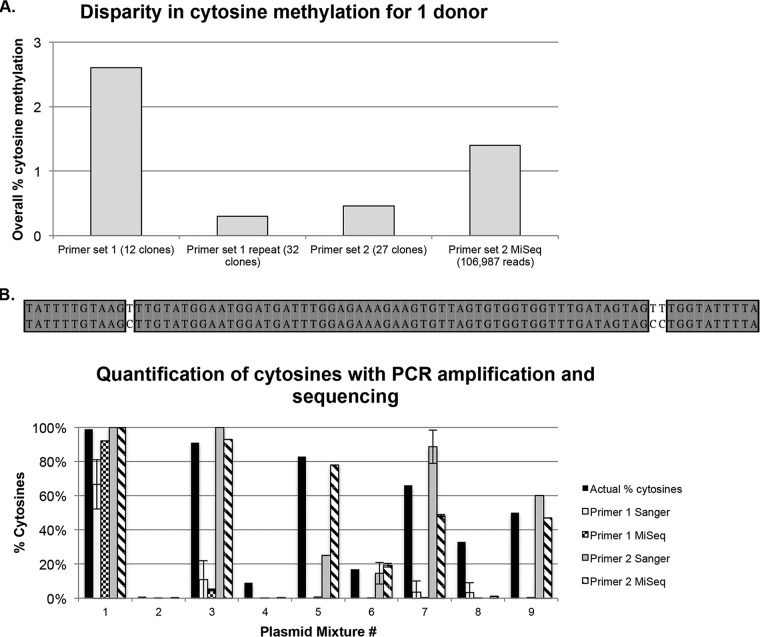
PCR amplification yields inconsistent results for % cytosines in the HIV LTR. (A) Intraexperimental variation of HIV methylation based on bisulfite sequencing of the LTR in one clinical sample. DNA was extracted from peripheral blood mononuclear cells (PBMCs) and bisulfite converted using the Zymo DNA Lightning kit. The proximal LTR was amplified with nested PCR using donor-specific primers, with variation in the nested forward primer only between experiments (primer set 1 versus 2). MiSeq was also conducted on the amplicon from primer set 2. Results from each experiment are reported for 73 cytosines across the amplicon. (B) Quantification of cytosines from PCR amplification and sequencing was performed from mixtures of two HIV LTR plasmids with variation at 3 positions (upper panel). PCR was performed with 2 different forward primers and subjected to MiSeq or cloning and Sanger sequencing. Quantification of cytosines from each condition was performed and compared to the known % cytosines (lower panel).

Multiple rounds of PCR can introduce stochastic bias in the presence of multiple variants (24); thus, it is difficult to measure methylation accurately in such cases. Indeed, PCR amplification of the HIV LTR from clinical samples using the same conditions can yield variable results across experiments, even with next-generation sequencing (NGS) technologies (Fig. 4A). Further, experiments to PCR amplify clones with mixed cytosine quantities showed that with both Sanger sequencing and NGS, percent methylation is neither accurate nor reproducible (Fig. 4B). This highlights the need for a different method for methylation quantification of the HIV provirus.

Bisulfite conversion and amplification of autosomal genes begin with much larger amounts of template, thus reducing the rounds of amplification required for successful cloning and sequencing. Because HIV is present in less than 100 copies per 100,000 CD4^+^ T cells (25), studying HIV DNA methylation necessitates more rounds of amplification. Additionally, autosomal genes are far less variable and in a static location compared to integrated HIV provirus. These technological hurdles call into question the usefulness of methylation quantification of the HIV provirus with this classical method.

### Sampling biases in bisulfite sequencing of HIV.

Chavez et al. (10) set a standard to examine a minimum of 9 clones when performing bisulfite sequencing on the HIV LTR. With few exceptions (6), published HIV methylation studies using clinical samples have examined <20 clones for most individuals (3–5). However, PLWH on long-term ART averaged 15 different HIV DNA sequences per individual (26). Further, the vast majority of the proviral populations in these individuals (i.e., 98%) were composed of defective sequences (26). As a consequence, this low level of sampling will not suffice to interrogate all proviral copies present in clinical samples, and because only a small subset of HIV DNA is replication competent, the majority of the data from clinical samples are from defective proviruses. Statistically, it would require at least 50 clones per individual to obtain at least one sequence from an intact provirus.

It is therefore not surprising that functional correlations with plasma HIV RNA were not evident in most cases, and cell-associated HIV RNA might be a better correlative measure. Alternatively, NGS offers much higher sampling depth than cloning and Sanger sequencing, yet studies using NGS for HIV methylation analysis have not made their way into the literature.

## CONCLUSIONS

The field of epigenetics is still new and requires more work before we can fully understand the effect of DNA methylation even upon autosomal genes. Because the integrated HIV provirus is subject to its immediate chromatin environment, determining the influence of cytosine methylation on the provirus *in vivo* is not a simple endeavor, and intraexperimental variation needs to be normalized. Emerging technologies and NGS should allow us to make headway in this area. Regardless, non-CpG methylation of the provirus has been ignored and should not be discounted during analysis of these data. Studies that rely on PCR amplification of the HIV provirus from bisulfite-converted samples should also be reproducible across multiple experiments and with multiple primer sets, and these replicates should be included with the primary data in future publications.

10.1128/mBio.02268-18.1 TEXT S1 Supplemental methods. Download Text S1, DOCX file, 0.2 MB. Copyright © 2019 LaMere et al. 2019 LaMere et al. This content is distributed under the terms of the Creative Commons Attribution 4.0 International license.

10.1128/mBio.02268-18.3 TABLE S1 Donor-specific primers used for amplification of the bisulfite converted HIV LTR. Download Table S1, DOCX file, 0.1 MB. Copyright © 2019 LaMere et al. 2019 LaMere et al. This content is distributed under the terms of the Creative Commons Attribution 4.0 International license.

10.1128/mBio.02268-18.4 TABLE S2 Primers used for amplification of the HIV LTR from each donor. Download Table S2, DOCX file, 0.01 MB. Copyright © 2019 LaMere et al. 2019 LaMere et al. This content is distributed under the terms of the Creative Commons Attribution 4.0 International license.

## References

[1] Tripathy MK, Abbas W, Herbein G 2011 Epigenetic regulation of HIV-1 transcription. Epigenomics 3:487–502. doi:10.2217/epi.11.61. 22126207

[2] Kauder SE, Bosque A, Lindqvist A, Planelles V, Verdin E 2009 Epigenetic regulation of HIV-1 latency by cytosine methylation. PLoS Pathog 5:e1000495. doi:10.1371/journal.ppat.1000495. 19557157 PMC2695767

[3] Blazkova J, Trejbalova K, Gondois-Rey F, Halfon P, Philibert P, Guiguen A, Verdin E, Olive D, Van Lint C, Hejnar J, Hirsch I 2009 CpG methylation controls reactivation of HIV from latency. PLoS Pathog 5:e1000554. doi:10.1371/journal.ppat.1000554. 19696893 PMC2722084

[4] Blazkova J, Murray D, Justement JS, Funk EK, Nelson AK, Moir S, Chun TW, Fauci AS 2012 Paucity of HIV DNA methylation in latently infected, resting CD4^+^ T cells from infected individuals receiving antiretroviral therapy. J Virol 86:5390–5392. doi:10.1128/JVI.00040-12. 22345448 PMC3347337

[5] Trejbalová K, Kovářová D, Blažková J, Machala L, Jilich D, Weber J, Kučerová D, Vencálek O, Hirsch I, Hejnar J 2016 Development of 5′ LTR DNA methylation of latent HIV-1 provirus in cell line models and in long-term-infected individuals. Clin Epigenetics 8:19. doi:10.1186/s13148-016-0185-6. 26900410 PMC4759744

[6] Weber S, Weiser B, Kemal KS, Burger H, Ramirez CM, Korn K, Anastos K, Kaul R, Kovacs C, Doerfler W 2014 Epigenetic analysis of HIV-1 proviral genomes from infected individuals: predominance of unmethylated CpG’s. Virology 449:181–189. doi:10.1016/j.virol.2013.11.013. 24418551 PMC4060985

[7] Palacios JA, Pérez-Piñar T, Toro C, Sanz-Minguela B, Moreno V, Valencia E, Gómez-Hernando C, Rodés B 2012 Long-term nonprogressor and elite controller patients who control viremia have a higher percentage of methylation in their HIV-1 proviral promoters than aviremic patients receiving highly active antiretroviral therapy. J Virol 86:13081–13084. doi:10.1128/JVI.01741-12. 22973038 PMC3497688

[8] Bednarik DP, Cook JA, Pitha PM 1990 Inactivation of the HIV LTR by DNA CpG methylation: evidence for a role in latency. EMBO J 9:1157–1164. doi:10.1002/j.1460-2075.1990.tb08222.x. 2323336 PMC551791

[9] Jones PA 2012 Functions of DNA methylation: islands, start sites, gene bodies and beyond. Nat Rev Genet 13:484–492. doi:10.1038/nrg3230. 22641018

[10] Chavez L, Kauder S, Verdin E 2011 In vivo, in vitro, and in silico analysis of methylation of the HIV-1 provirus. Methods 53:47–53. doi:10.1016/j.ymeth.2010.05.009. 20670606 PMC3566233

[11] Kypr J, Mrazek J, Reich J 1989 Nucleotide composition bias and CpG dinucleotide content in the genomes of HIV and HTLV 1/2. Biochim Biophys Acta 1009:280–282. doi:10.1016/0167-4781(89)90114-0. 2597678

[12] Alinejad-Rokny H, Anwar F, Waters SA, Davenport MP, Ebrahimi D 2016 Source of CpG depletion in the HIV-1 genome. Mol Biol Evol 33:3205–3212. doi:10.1093/molbev/msw205. 27682824

[13] Wang D, Liu X, Zhou Y, Xie H, Hong X, Tsai HJ, Wang G, Liu R, Wang X 2012 Individual variation and longitudinal pattern of genome-wide DNA methylation from birth to the first two years of life. Epigenetics 7:594–605. doi:10.4161/epi.20117. 22522910 PMC3398988

[14] Yan J, Zierath JR, Barres R 2011 Evidence for non-CpG methylation in mammals. Exp Cell Res 317:2555–2561. doi:10.1016/j.yexcr.2011.08.019. 21925168

[15] Jang HS, Shin WJ, Lee JE, Do JT 2017 CpG and Non-CpG methylation in epigenetic gene regulation and brain function. Genes (Basel) 8:148. doi:10.3390/genes8060148. PMC5485512 28545252

[16] Patil V, Ward RL, Hesson LB 2014 The evidence for functional non-CpG methylation in mammalian cells. Epigenetics 9:823–828. doi:10.4161/epi.28741. 24717538 PMC4065179

[17] Peng X, Sova P, Green RR, Thomas MJ, Korth MJ, Proll S, Xu J, Cheng Y, Yi K, Chen L, Peng Z, Wang J, Palermo RE, Katze MG 2014 Deep sequencing of HIV-infected cells: insights into nascent transcription and host-directed therapy. J Virol 88:8768–8782. doi:10.1128/JVI.00768-14. 24850744 PMC4136300

[18] Fang JY, Mikovits JA, Bagni R, Petrow-Sadowski CL, Ruscetti FW 2001 Infection of lymphoid cells by integration-defective human immunodeficiency virus type 1 increases de novo methylation. J Virol 75:9753–9761. doi:10.1128/JVI.75.20.9753-9761.2001. 11559808 PMC114547

[19] Mikovits JA, Young HA, Vertino P, Issa JP, Pitha PM, Turcoski-Corrales S, Taub DD, Petrow CL, Baylin SB, Ruscetti FW 1998 Infection with human immunodeficiency virus type 1 upregulates DNA methyltransferase, resulting in de novo methylation of the gamma interferon (IFN-gamma) promoter and subsequent downregulation of IFN-gamma production. Mol Cell Biol 18:5166–5177. doi:10.1128/MCB.18.9.5166. 9710601 PMC109102

[20] Lorincz MC, Schubeler D, Goeke SC, Walters M, Groudine M, Martin DI 2000 Dynamic analysis of proviral induction and de novo methylation: implications for a histone deacetylase-independent, methylation density-dependent mechanism of transcriptional repression. Mol Cell Biol 20:842–850. doi:10.1128/MCB.20.3.842-850.2000. 10629041 PMC85201

[21] Dodge JE, Ramsahoye BH, Wo ZG, Okano M, Li E 2002 De novo methylation of MMLV provirus in embryonic stem cells: CpG versus non-CpG methylation. Gene 289:41–48. doi:10.1016/S0378-1119(02)00469-9. 12036582

[22] Cuevas JM, Geller R, Garijo R, López-Aldeguer J, Sanjuán R 2015 Extremely high mutation rate of HIV-1 in vivo. PLoS Biol 13:e1002251. doi:10.1371/journal.pbio.1002251. 26375597 PMC4574155

[23] Shen L, Guo Y, Chen X, Ahmed S, Issa JP 2007 Optimizing annealing temperature overcomes bias in bisulfite PCR methylation analysis. Biotechniques 42:48–50. doi:10.2144/000112312. 17269485

[24] Best K, Oakes T, Heather JM, Shawe-Taylor J, Chain B 2015 Computational analysis of stochastic heterogeneity in PCR amplification efficiency revealed by single molecule barcoding. Sci Rep 5:14629. doi:10.1038/srep14629. 26459131 PMC4602216

[25] Eriksson S, Graf EH, Dahl V, Strain MC, Yukl SA, Lysenko ES, Bosch RJ, Lai J, Chioma S, Emad F, Abdel-Mohsen M, Hoh R, Hecht F, Hunt P, Somsouk M, Wong J, Johnston R, Siliciano RF, Richman DD, O’Doherty U, Palmer S, Deeks SG, Siliciano JD 2013 Comparative analysis of measures of viral reservoirs in HIV-1 eradication studies. PLoS Pathog 9:e1003174. doi:10.1371/journal.ppat.1003174. 23459007 PMC3573107

[26] Bruner KM, Murray AJ, Pollack RA, Soliman MG, Laskey SB, Capoferri AA, Lai J, Strain MC, Lada SM, Hoh R, Ho YC, Richman DD, Deeks SG, Siliciano JD, Siliciano RF 2016 Defective proviruses rapidly accumulate during acute HIV-1 infection. Nat Med 22:1043–1049. doi:10.1038/nm.4156. 27500724 PMC5014606

[27] Barbato G, Barini EM, Genta G, Levi R 2011 Features and performance of some outlier detection methods. J Appl Stat 38:2133–2149. doi:10.1080/02664763.2010.545119.

