# Supplementary figures and images for: Challenges in Quantifying Cytosine Methylation in the HIV Provirus
Source: mBio. 2019 Jan 22;10(1):e02268-18. doi: 10.1128/mBio.02268-18 (PMC6343035; doi:10.1128/mBio.02268-18)

**A.**

Unconverted cytosines across lambda control

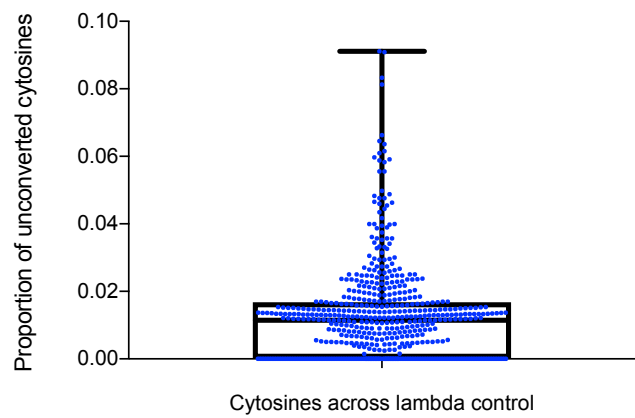

**B.**

Cytosine Conversion in Lambda Control

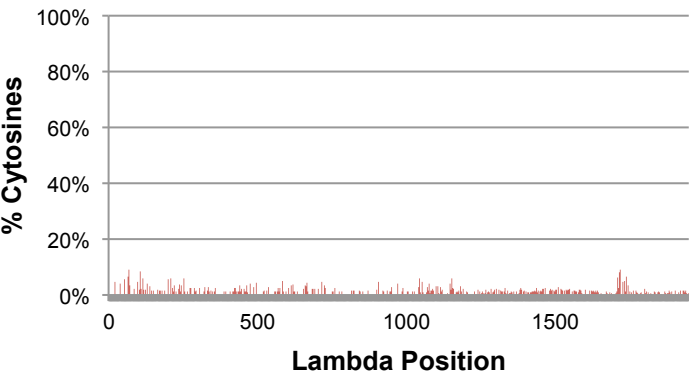

**C.**

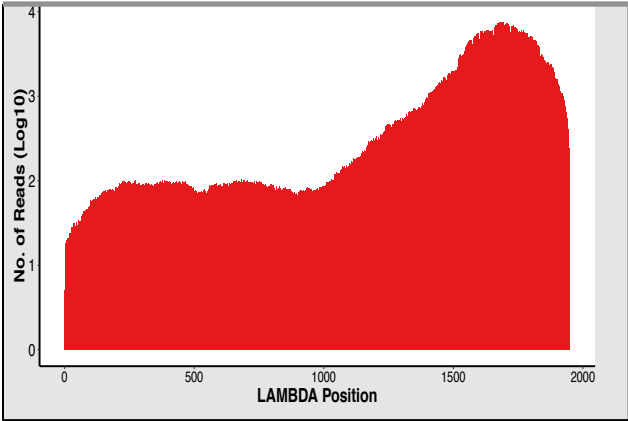

**D.**

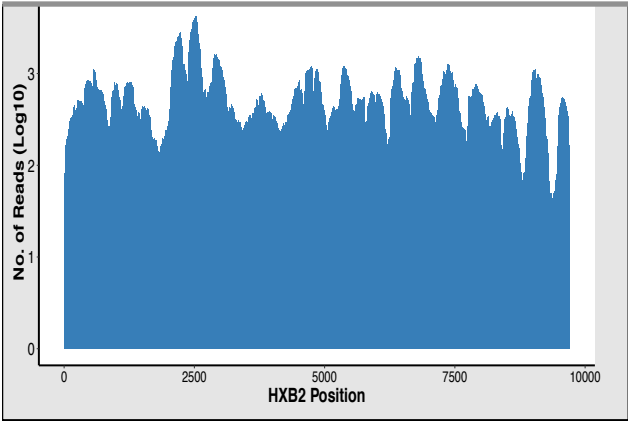

Supplement: FIG S1 [file mBio.02268-18-sf001.pdf]
