# Supplementary material for: Challenges in Quantifying Cytosine Methylation in the HIV Provirus
Source: mBio. 2019 Jan 22;10(1):e02268-18. doi: 10.1128/mBio.02268-18 (PMC6343035; doi:10.1128/mBio.02268-18)
Supplement: TEXT S1 [file mBio.02268-18-s0001.docx]

**Text S1: Methods**

**Ethics Statement**

All the studies in this manuscript were covered by Human Subjects Research Protocols approved by the Institutional Review Board of The University of California San Diego. Informed written consent was obtained from all study subjects in the study.

**Study Population and Samples**

Samples from seven individuals in the California Collaborative Treatment Group (CCTG) and seven individuals from the San Diego Primary Infection Resource Consortium were included in this study. All samples were collected from suppressed individuals (<50 viral copies/mL plasma) taking antiretroviral therapy.

**DNA Extraction**

DNA purification was performed on PBMCs from a total of 14 donors receiving ART. DNA was purified using the Qiagen DNEasy Blood and Tissue kit and quantitated using Nanodrop spectrophotometer.

**Whole genome bisulfite sequencing:**

One ug of PBMC genomic DNA spiked with lambda DNA was sheared in 52.5 uL of Qiagen buffer EB with a Covaris m220 ultrasonicator with the following parameters: Peak Power 75.0, Duty Factor 20.0, Cycles/Burst 200, Duration 150 sec in 30 sec increments. Following shearing, DNA was end repaired and A-tailed using the KAPA HyperPrep kit per manufacturing instructions. A 30 minute ligation was performed to adapters from the Roche SeqCap Adapter Kit A per manufacturer instructions using the KAPA HyperPrep kit. Ligated DNA was purified with 88 uL of Ampure beads per manufacturer instructions and eluted in 100 uL of Qiagen buffer EB. DNA was then size selected using SPRI dual selection with the following protocol: 70 uL of Ampure beads were added to 100 uL of ligated DNA and incubated for 5 minutes. 165 uL of supernatant was transferred to a new tube, and 20 uL of Ampure beads was added to the DNA and incubated for 5 minutes. Ampure bead purification was performed per manufacturer instructions and eluted in 20 uL of Qiagen buffer EB. Bisulfite conversion was performed using the Zymo EZ DNA Methylation-Lightning kit per manufacturer instructions and eluted in 20 uL of Zymo elution buffer into low bind 1.5 mL tubes. Ligation-mediated PCR was performed using the KAPA HiFi HotStart Uracil+ ReadyMix and the KAPA library amplification primer mix per manufacturer instructions in a 50 uL reaction. Amplified bisulfite-converted DNA was purified using 70 uL of Ampure beads per manufacturer instructions and eluted in 50 uL of PCR-grade water. Purified DNA was analyzed on an Agilent TapeStation to validate correct sizing, and 20 pmol of DNA was loaded with 20% PhiX onto an Illumina MiSeq instrument using the MiSeq Reagent Kit V3 600-cycle paired end sequencing kit.

**HIV LTR Bisulfite PCR and plasmid PCR**

2 ug of PBMC genomic DNA was bisulfite converted using the Zymo EZ DNA Methylation-Lightning kit per manufacturer instructions and eluted in 20 uL of elution buffer. For PCR, 120 ng of bisulfite converted DNA was subjected to nested PCR using donor-specific primers designed from unconverted sequences **(Supplementary Table I and II)**. Second round PCR included MiSeq adapters tagged onto the 5’ end of the sequence-specific primers. Both rounds of PCR were performed using Invitrogen Platinum Taq DNA Polymerase with 1.5 mM MgCl2, 200 uM dNTPs, and 200 nM of each primer. Reactions were performed in an Applied Biosystems Veriti thermocycler with the following program: 94° for 2 min., 40 cycles of 94° for 15 sec., 55° for 90 sec., and 72° for 1 min., 72° for 10 min. Second round PCR was performed with 2 uL of the first round reaction. GAPDH controls were amplified with 40 cycles using the same program and purified. 10 uL of PCR product was run on a 1% TAE agarose gel with ethidium bromide and gel purified using the Qiagen Qiaquick PCR gel purification kit per manufacturer instructions. Products were eluted in 30 uL of Buffer EB. For plasmid DNA mixtures, 100 copies of plasmid were amplified using nested PCR with M13 primers for first round and sequence-specific primers for second round. First round PCR with M13 primers was performed with the conditions above, but with a 50° annealing temperature.

I**llumina Seqencing of PCR products**

TruSeq Indices were added to 5 uL of purified PCR products using KAPA HiFi HotStart ReadyMix according to manufacturer instructions, purified using 56 uL of Ampure Beads, and eluted in 25 uL of Tris pH 8.5. Purified DNA was analyzed on an Agilent TapeStation to validate correct sizing, and 20 pmol of DNA was loaded with 20% PhiX onto an Illumina MiSeq instrument using the MiSeq Reagent Kit V3 600-cycle paired end sequencing kit.

**NGS Sequence Analysis**

For both whole genome sequencing and PCR products, reads with PHRED quality above or equal to 30 were mapped to the HXB2 reference genome with SMALT mapping tool ([https://www.sanger.ac.uk/science/tools/smalt-0](https://hsmail.ucsd.edu/owa/redir.aspx?C=-gF8RothGNztlnZqH2tRNJ8YSW51tMSRlTDf85B05KOyf5DXGDHWCA..&URL=https%3a%2f%2fwww.sanger.ac.uk%2fscience%2ftools%2fsmalt-0)) Reads for each nucleotide position were calculated and filtered with a threshold of 20 reads or more. Percent cytosine methylation for each cytosine position was calculated based on number of reads containing cytosine divided by total number of reads containing both cytosine and thymine.

**Cloning and Sanger Sequencing**

PCR products were cloned using the Thermo-Fisher Topo TA Cloning kit, and plasmids were transformed into Thermo-Fisher Top10 cells. Transformed competent cells were grown at 37 degrees on 50 ug/mL carbenicillin LB plates. Nine to 10 colonies were picked for each PCR product, grown in 2 mL of LB 50 ug/mL carbenicillin, and purified using the Qiagen MiniPrep kit. Purified clones were sequenced with with M13F using dye terminator sequencing on an ABI prism 3100 genetic analyzer.

**Sanger Sequence Analysis**

Electrophoretograms were trimmed for vector and primers, and sequences were aligned using MacVector software. Percent cytosine methylation was calculated for each cytosine position based on number of cytosines divided by total cytosines and thymines.
