# Supplementary material for: Challenges in Quantifying Cytosine Methylation in the HIV Provirus
Source: mBio. 2019 Jan 22;10(1):e02268-18. doi: 10.1128/mBio.02268-18 (PMC6343035; doi:10.1128/mBio.02268-18)
Supplement: TABLE S1 [file mBio.02268-18-st001.docx]

| **Supplementary Table I:** Donor-specific primers used for amplification of the bisulfite converted HIV LTR. | |
| --- | --- |
| **Name** | **Sequence** |
| 118F | TATTGATTTTTGGATGGTGTTWTAAGTTAG |
| 118FB | TATTGATTTTTGGGTGGTGTTTTAAGTTAG |
| 118FC | TATTGATTTTTGGITRGTGTTTTAAGTTAG |
| 118FD | TATTGTGTTTTRGITGGTGTTTTAAGTTAG |
| 118FE | TTTTGATTTTTGGITRGTGTTTTAAGTTAG |
| 506R | CCTAATTAACCAAAAAACTCCCAAAC |
| 506RB | CCTAATCAACCAAAAAACTCCCAAAC |
| 506RC | TCCCTAACYAACCAAAAAACTCCCAAAC |
| 506RD | TCCCTAAATAACCAAAAAACTCCCAAAC |
| 504RE | TCCTTAACTAACCAAAAAACTCCYAAAC |
| 575R | CACAAYAAACRAACACACACTAC |
| 124FMISQ | TCGTCGGCAGCGTCAGATGTGTATAAGAGACAGTTTTTGGITRGTGTTTTAAGTTAGTATTAG |
| 124FBMISQ | TCGTCGGCAGCGTCAGATGTGTATAAGAGACAGGTTTTRGITGGTGTTTTAAGTTAGTATTAG |
| 128FMISQ | TCGTCGGCAGCGTCAGATGTGTATAAGAGACAGTGGATGGTGTTWTAAGTTAGTRTTAGTTG |
| 128FBMISQ | TCGTCGGCAGCGTCAGATGTGTATAAGAGACAGTTRGITGGTGTTTTAAGTTAGTATTATTTG |
| 485RMISQ | GTCTCGTGGGCTCGGAGATGTGTATAAGAGACAGCAAACTCAAATCTAATCTAACCAAAAAAAC |
| 485RBMISQ | GTCTCGTGGGCTCGGAGATGTGTATAAGAGACAGCAAACTCAAATCTAAWCTAACCAAAAAAAC |
| 485RCMISQ | GTCTCGTGGGCTCGGAGATGTGTATAAGAGACAGCYAAACTCAAATCTAATCTAACMAAAAAAAC |
| 485RDMISQ | GTCTCGTGGGCTCGGAGATGTGTATAAGAGACAGCAAACTCWAATCTAATCTAMCCAAAAAAAC |
| GAPDH-MISQF | TCGTCGGCAGCGTCAGATGTGTATAAGAGACAGGTGTGGGAGGAGTTATTTGGTTG |
| GAPDH-MISQR | GTCTCGTGGGCTCGGAGATGTGTATAAGAGACAGCAAAACTAAATCAACTTCCCCTCCC |
