# Supplementary material for: Challenges in Quantifying Cytosine Methylation in the HIV Provirus
Source: mBio. 2019 Jan 22;10(1):e02268-18. doi: 10.1128/mBio.02268-18 (PMC6343035; doi:10.1128/mBio.02268-18)
Supplement: TABLE S2 [file mBio.02268-18-st002.docx]

| **Supplementary Table II:** Primers used for amplification of the HIV LTR from each donor. | | | | |
| --- | --- | --- | --- | --- |
| **Donor** | **1st round forward primer** | **1st round reverse primer** | **2nd round forward primer** | **2nd round reverse primer** |
| TD1 | 118FE | 504RE | 128F | 485R |
| TD2 | 118FC | 506RC | 124F | 485R |
| CD1 | 118F | 506R | 128F | 485R |
| CD2 | 118F | 506R | 128F | 485R |
| CD3 | 118FB | 506R | 124F | 485RD |
| CD5 | 118FC | 506R | 124F | 485R |
| C1 | 118FC | 506RC | 124F | 485RB |
| C3 | 118FC | 506RB | 124F | 485RB |
| C4 | 118FC | 506RB | 124F | 485RB |
| C6 | 118FC | 506RD | 124F | 485RD |
| C7 | 118FE | 575R | 124F | 485RC |
| C9 | 118FD | 575R | 124FB | 485RC |
| C10 | 118FD | 506RC | 128FB | 485RD |
